# A QM/MM study of the nature of the entatic state in plastocyanin

**DOI:** 10.1002/jcc.24666

**Published:** 2016-11-14

**Authors:** Catherine A. Hurd, Nicholas A. Besley, David Robinson

**Affiliations:** ^1^ School of ChemistryUniversity of NottinghamUniversity ParkNottinghamNG7 2RDUnited Kingdom

**Keywords:** QM/MM, electron transfer, plastocyanin

## Abstract

Plastocyanin is a copper containing protein that is involved in the electron transfer process in photosynthetic organisms. The active site of plastocyanin is described as an entatic state whereby its structure represents a compromise between the structures favored by the oxidized and reduced forms. In this study, the nature of the entatic state is investigated through density functional theory‐based hybrid quantum mechanics/molecular mechanics (QM/MM) molecular dynamics simulations. The strain energy is computed to be 12.8 kcal/mol and 14.5 kcal/mol for the oxidized and reduced forms of the protein, indicating that the active site has an intermediate structure. It is shown that the energy gap between the oxidized and reduced forms varies significantly with the fluctuations in the structure of the active site at room temperature. An accurate determination of the reorganization energy requires averaging over conformation and a large region of the protein around the active site to be treated at the quantum mechanical level. © 2016 The Authors. Journal of Computational Chemistry Published by Wiley Periodicals, Inc.

## Introduction

Copper containing proteins are naturally occurring proteins involved in many biological processes, including respiration and photosynthesis.^1^, ^2^, ^3^, ^4^, ^5^, ^6^, ^7^, ^8^, ^9^ One of the most prominent examples of these proteins is plastocyanin which transfers an electron from the cytochrome *bf* complex to photosystem I in photosynthetic organisms. In the active site of plastocyanin, copper is bound in an approximate trigonal plane to the sulphur of cysteine and two nitrogen atoms of histidine ligands, with a further methionine ligand in an axial position.

During electron transfer copper is reduced from Cu(II) to Cu(I). In its oxidized form, plastocyanin has a d^9^ electronic configuration where the singly occupied molecular orbital (SOMO) corresponds to an antibonding combination of a copper 3d orbital and a 3p orbital on the sulphur atom of the coordinated cysteine ligand.^10^ In this state, the protein has a rich spectroscopy, and has been studied by several different spectroscopic techniques including electronic absorption, circular dichroism, magnetic circular dichroism, and x‐ray absorption spectroscopies.^11^, ^12^ Alongside these experimental measurements there have been numerous theoretical studies with a wide variety of different levels of approximation.^7^, ^13^, ^14^, ^15^, ^16^, ^17^, ^18^, ^19^, ^20^, ^21^, ^22^, ^23^, ^24^ These studies have led to an understanding of the nature of the observed spectroscopic features and detailed insight into the electronic structure of the active site. For example, the absorption spectrum shows peaks at 16,700 cm^−1^ and 12,800 cm^−1^, which are assigned to a Cys(π) → SOMO ligand to metal charge‐transfer (LMCT) and d ligand field → SOMO excitations, respectively, the first of which is much more intense than the second.^7^ At higher energy (21,400 cm^−1^) there is an additional band which is assigned to the Cys(σ) → SOMO transition. In plastocyanin this band is very weak, but in the closely related proteins cucumber basic protein and nitrite reductase its intensity increases.^12^, ^25^ This change can be associated with changes in the structure of the active site,^10^ and other work has shown the excitation energy of the intense LMCT band correlate strongly with the copper‐cysteine bond length.^14^ The spectroscopy of the reduced form of the protein contains less information, thus there have been fewer studies that have focused on this form of the protein.^26^, ^27^, ^28^

The structure of the active site plays a key role in the function of the protein. One debated aspect of plastocyanin redox chemistry has been the entatic^29^ (or reduced rack^30^) state, whereby the structure of the active site represents a compromise between the structures favored by the oxidized and reduced forms of the protein. Adopting such an intermediate structure leads to fast electron transfer rates since the reorganization energy will be small. In experiment the reorganization energy has been measured to be 16–28 kcal/mol.^31^, ^32^, ^33^ It is thought that the structure for the active site is a consequence of the extended structure of the protein. Indeed, it has been shown with other protein systems that the protein holds transition metal active sites in such a geometry that the conversion between electronic or spin states requires much lower energies than would be the case for the equivalent “model system” of the active site.^34^

This concept of an entatic state was questioned following quantum mechanical calculations of the structure of the active site.^35^, ^36^, ^37^ In this work, the structure of the active site was optimized free from the constraints of the protein using density functional theory (DFT) and shown to have a very similar structure to the crystal structure. It was concluded that the active site was not strained to a significant extent in either the oxidized and reduced forms of the protein.^37^ However, studies of the isolated active site with relatively small model systems will only probe any strain that is localized very near the copper center. These studies initiated further discussion of the copper active site. Further analysis of experimental evidence was found to indicate that the active site of blue copper proteins is constrained for functional advantage with a low reorganization energy for electron transfer.^38^, ^39^ Deeth studied the oxidized form of a range of blue copper proteins, including plastocyanin, with ligand field molecular mechanics (LFMM) and found an entatic strain energy of about 10 kcal mol^−1^.^18^ Further hybrid quantum mechanics/molecular mechanics (QM/MM) approaches^24^ and implicit models of the protein environment^40^ have been used to obtain optimized geometries of each state.

The studies of the strain energy and the reorganization energy have been largely based on a static view of the structures. It is known that conformational averaging is important in determining the properties of these proteins.^16^, ^23^ In this article, we develop a more dynamic picture of the electron transfer process. Previous work has studied photoinduced electron transfer dynamics between the oxidized ground state and optically excited LMCT state based on classical molecular dynamics (MD) simulations.^41^ The parameterization of classical force fields to accurately describe the structure of the active site is problematic, and a previous study has shown that electronic absorption and circular dichroism spectra computed from structures from a classical MD trajectory were in poor agreement with experiment compared with those based on structures from a LFMM‐based MD simulation.^23^ QM/MM approaches have the potential to provide a more accurate and balanced treatment of the oxidized and reduced forms of the protein. However, the size of the QM region in these calculations can be critical and there needs to be a balance between the computational cost of a large QM region and the length of MD simulation that can be performed. Here, we investigate the dynamics and the associated reorganization energy of the oxidized and reduced forms of plastocyanin using a QM/MM approach combining DFT with an all‐electron basis set for the active site with the remainder of the protein described by a classical force field. The importance in the choice of the size of the active site treated at the QM level is explored with further calculations with a very large QM region.

## Methods

### Gas‐phase quantum chemical calculations

**Figure 1:**
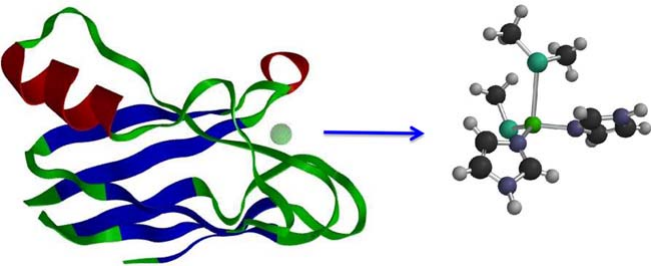
Plastocyanin and the model of the active site used in the QM calculations. [Color figure can be viewed at wileyonlinelibrary.com]

The structure of the gas‐phase model active site (see Fig. 1) was optimized with DFT with the ωB97X‐D functional^42^, ^43^ and 6‐31G(d)^44^, ^45^, ^46^ basis set for both the Cu(I) and Cu(II) oxidation states. The ωB97X‐D functional incorporates an atom–atom dispersion term and has been shown to be accurate for a wide range of applications including thermochemistry, fundamental gaps, and noncovalent interactions which are relevant for this study.^43^ Harmonic frequency calculations were performed to ensure the resulting structures corresponded to energy minima.

### QM/MM dynamics

Initially, reduced and oxidized forms of plastocyanin from the Dryopteris fern (PDB codes 1KDI and 1KDJ, respectively)^47^ were minimized and equilibrated using the CHARMM36 protein force‐field,^48^, ^49^ with additional bonding terms for the active site taken from the work of Voth and coworkers,^41^ the partial atomic charges from the CHARMM36 force‐field were retained, while the copper center was assigned the formal charge relevant to the oxidation state. The system was created using the zwitterionic form of plastocyanin and 5496 TIP3P^50^ water molecules, using a truncated octahedral symmetry. Nineteen K^+^ and thirteen Cl^−^ ions were added to neutralize the overall charge of the system, giving a physiologically relevant concentration of 0.15 M. Periodic boundaries were employed, using the Particle‐mesh Ewald summation method for long‐range electrostatics, with a cutoff distance of 12 Å and a pair‐list distance of 16 Å. Minimization was performed using the steepest‐descent algorithm for 10,000 steps. Equilibration was carried out for a total of 1 ns, with a timestep of 2 fs, with rescaling of velocities every 500 steps. All bonds to hydrogen were constrained using the SHAKE algorithm. This gave an equilibrated protein and water molecules system that provides a suitable starting point for further calculations.

Using the equilibrated system, a QM/MM partitioning was performed, using the active site shown in Figure 1, with hydrogen link‐atoms where the bond was broken for His37, Cys87, His90, and Met95, giving a total of 33 QM atoms (identical to the atoms used in the gas‐phase calculations). PME was used only for the MM/MM partition, with QM/MM polarization for the QM region. For the case of electron transfer, long‐range electrostatics maybe important and thus the QM/MM approach may be limited. For both the oxidized and reduced forms, QM/MM minimization was performed until the energy change was less than 1 × 10^−4^ kcal mol^−1^, before a short period of equilibration was performed (using the same parameters as above; a total of 2 ps). Production QM/MM dynamics were performed for a total of 40 ps. The ωB97X‐D functional and the 6‐31G(d) basis set were used throughout. All of the quantum chemical calculations were performed with the Q‐Chem software,^51^ while the QM/MM simulations were performed with the Q‐Chem/CHARMM interface,^52^, ^53^ using the CHARMM36 protein force‐field and the TIP3P water model. Further QM calculations were performed with a greatly enlarged QM region that comprised 1144 atoms, chosen by including all residues which are within (or have at least one atom within) a sphere of radius 9 Å of the copper centre, no MM charges were included in these calculations. For these calculations the 6‐31G* basis set was used for the copper and cysteine sulphur atoms, while the 6‐31G basis set was used for the remaining atoms. Overall this gave a calculation with nearly 6000 basis functions.

## Results and Discussion

Tables 1 and 2 summarize the values of key geometrical parameters of the active site for both the Cu(II) (oxidized) and Cu(I) (reduced) model compounds. Results are shown for the isolated active site (QM atoms only, no MM charges), average values from the QM/MM simulations and from the crystal structure. Several other computational studies have investigated the structure of the active site of the oxidized and reduced forms. Pavelka and Burda^40^ found optimized structures of the active site in gas‐phase and with implicit solvent models^39^ to differ significantly from the crystal structure, particularly for the Cu—S(met) bond length for both oxidized and reduced forms of the protein. Relative to the crystal structure, they found this bond length was reduced significantly in the model active sites for the oxidized form while a very large increase was observed for the reduced form to give an unusually large (essentially not bonded) bond length. The results presented here show a significant decrease in the Cu—S(met) bond length for both the oxidized and reduced forms of the protein with respect to the crystal structures, which is consistent with other studies.^17^, ^37^ Overall, the structures for the active site in gas‐phase show significant differences from the crystal structure for both the bond lengths and dihedral angles to the coordinating ligands.

**Table 1:** Structural parameters for the oxidized form of plastocyanin.

Geometrical parameters	Active site	QM/MM	Crystal structure^[a]^
**Bonds**			
Cu—S(cys)	2.18	2.16 (0.05)	2.26
Cu—S(met)	2.36	2.78 (0.16)	2.92
Cu—N(his37)	2.00	1.95 (0.05)	1.93
Cu—N(his90)	1.98	1.91 (0.04)	2.07
**Angles**			
S(cys)—Cu—S(met)	107.5	102.7 (4.4)	106.7
N(his37)—Cu—N(his90)	97.0	99.5 (3.2)	107.3
S(cys)—Cu—N(his37)	140.1	129.7 (5.3)	126.1
S(cys)—Cu—N(his90)	96.5	122.1 (7.3)	118.3
**Dihedral Angles**			
C_β_(cys)—S(cys)—Cu—S(met)	41.5	−12.4 (6.8)	−8.9
C_γ_(his37)—N(his37)—Cu—S(met)	22.7	−117.6 (9.0)	−118.6
C_γ_(his90)—N(his90)—Cu—S(met)	88.6	55.3 (9.0)	47.4
**Improper Torsions**			
Cu—S(cys)—N(his90)—N(his37)	−24.2	−19.1 (5.8)	−25.2
Cu—S(cys)—N(his37)—N(his90)	53.3	22.1 (7.8)	33.1
Cu—N(his90)—N(his37)—S(cys)	−25.5	−15.0 (4.8)	−17.1

Bond lengths in Å and angles in degrees. QM/MM values correspond to average values over the simulation with root mean squared variations given in parenthesis.

a Crystal structure with a resolution of 1.7 Å from Ref. [ 47]

**Table 2:** Structural parameters for the reduced form of plastocyanin.

Geometrical parameters	Active site	QM/MM	Crystal structure^[a]^
**Bonds**			
Cu—S(cys)	2.32	2.16 (0.06)	2.21
Cu—S(met)	2.30	2.63 (0.29)	2.91
Cu—N(his37)	2.00	1.97 (0.08)	1.95
Cu—N(his90)	1.98	1.92 (0.08)	2.10
**Angles**			
S(cys)—Cu—S(met)	113.8	107.1 (6.8)	108.2
N(his37)—Cu—N(his90)	118.3	100.6 (5.8)	104.6
S(cys)—Cu—N(his37)	103.9	129.4 (8.4)	130.1
S(cys)—Cu—N(his90)	108.8	112.8 (10.2)	117.3
**Dihedral Angles**			
C_β_(cys)—S(cys)—Cu—S(met)	−40.1	−6.1 (10.2)	−3.8
C_γ_(his37)—N(his37)—Cu—S(met)	−110.2	−131.5 (10.2)	−114.6
C_γ_(his90)—N(his90)—Cu—S(met)	134.5	54.2 (11.6)	51.7
**Improper Torsions**			
Cu—S(cys)—N(his90)—N(his37)	−31.3	−25.2 (8.4)	−17.6
Cu—S(cys)—N(his37)—N(his90)	29.7	33.1 (9.6)	22.8
Cu—N(his90)—N(his37)—S(cys)	−40.6	−22.6 (6.3)	−15.9

Bond lengths in Å and angles in degrees. QM/MM values correspond to average values over the simulation with root mean squared variations given in parenthesis.

a Crystal structure with a resolution of 1.8 Å from Ref. 47.

The average structural parameters from the QM/MM simulations are in closer agreement with the crystal structure, particularly considering some variation between the reported crystal structures and the dynamical nature of the protein in solution is to be expected. The predicted Cu—S(cys) bond length for the oxidized form of 2.16 Å is consistent with the value of 2.15 Å derived from analysis of the absorption spectrum^20^ and is also agrees with the value of 2.17 Å derived from LFMM‐based MD simulations.^18^ During the QM/MM simulations, the bond lengths typically fluctuate by about 0.05 Å. The exception to this is the Cu—S(met) bond length which has a significantly larger variation during the simulation, and the extent of this variation is found to be greater for the reduced form of the protein. This is consistent with the nature of the Cu—S(met) bond which is known to be a relatively facile bond. Similarly, a large variation in the dihedral angles involving the S(met) ligand are observed. The sensitivity of the Cu—S(met) bond length to the protein environment has been observed in previous studies. Sinnecker and Neese^17^ found that an isolated model active site was not able to reproduce the long crystal structure Cu—S(met) bond length, however, a much closer value was obtained in QM/MM calculations. Furthermore, Ryde et al. showed this bond length to increase with the value of dielectric constant in calculations with an implicit solvent model.^37^

The reduced form exhibits tetrahedral coordination to the copper center, while the oxidized form shows a more distorted geometry. For the oxidized form, it is useful to calculate the geometrical *τ*_4_ parameter,^54^ given as

(1) $$\tau_{4}=\frac{360{}^{\circ}-\left(\alpha +\beta \right)}{141{}^{\circ}}$$

where *α* and *β* are the two largest bond angles of the copper coordination site and the value of *τ*_4_ varies between 0 for square‐planar and 1 for tetrahedral geometries. For the isolated active site values of *τ*_4_ = 0.57 and *τ*_4_ = 0.91 are obtained for the oxidized and reduced forms. This shows that the reduced form is close to tetrahedral while the oxidized form is intermediate between square‐planar and tetrahedral; this is typical for 4‐coordinate Cu(II) systems. For the average structures from the QM/MM simulations the value of *τ*_4_ for the oxidized form increases to 0.88, while the reduced form has a value close to 1. Consequently, the distortion from tetrahedral evident in the model gas phase structure of the oxidized form is decreased significantly.

The strain energy provides a measure of the extent to which the protein holds the active site in an energetically nonoptimum structure, and is calculated from the difference between the energy of the ensemble averaged QM/MM active site structure in the gas‐phase (i.e., no MM charges included) and the energy of the corresponding optimized active‐site gas‐phase structure. This gives values of 12.8 kcal/mol and 14.5 kcal/mol for the oxidized and reduced forms of the protein, respectively. The value for the oxidized form is close to the value of ∼10 kcal/mol determined from LFMM calculations.^18^ Here, it is shown that the value for the reduced form has a similar magnitude to that of the oxidized form. In both cases, there is significant strain on the coordination center. These values are consistent with the premise of an entatic state where the geometry of the active site is intermediate between the optimal geometries of the two states.

**Figure 2:**
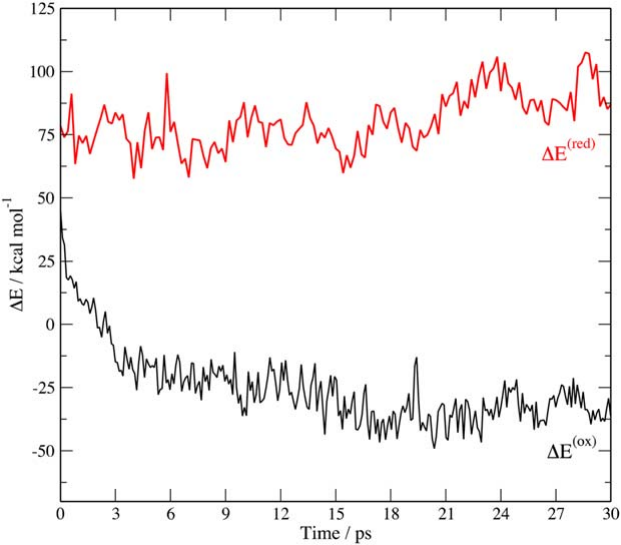
Variation in Δ*E* (*E*_ox_ − *E*_red_) during the QM/MM molecular dynamics simulations for the oxidized and reduced forms of the protein. [Color figure can be viewed at wileyonlinelibrary.com]

Figure 2 shows the variation in Δ*E*, where Δ*E* = *E*_ox_ − *E*_red_, during the QM/MM simulations for the oxidized and reduced forms of the protein. This corresponds to the energy change between the two states without allowing for any relaxation of the structure. For both trajectories there is significant variation in Δ*E*. For the reduced trajectory, the average value of Δ*E* is 82.5 kcal/mol with a standard deviation of 11.2 kcal/mol. The oxidized trajectory shows a surprising amount of deviation in Δ*E*, which is initially positive and then falls rapidly, where it varies predominantly between −10 kcal/mol and −40 kcal/mol. The initial rapid change in Δ*Ε* at the start of the simulation is likely to reflect that the protein was still equilibrating during this period. Focusing on the simulation from 3 ps onwards gives an average value for Δ*Ε* of −29.2 kcal/mol with a standard deviation of 9.0 kcal/mol. This variation in Δ*Ε* suggests that the view of the protein being held in a single entatic state is not the complete picture since an ensemble of structures with significantly different Δ*Ε* will be sampled.

The reorganization energy is a key component in Marcus theory where the rate of electron transfer is given by^55^

(2) $$k_{\text{ET}}=\frac{{\left|V\right|}^{2}}{\hbar }\sqrt{\frac{\pi }{\lambda k_{B}T}}\exp\left[-\frac{{\left(\lambda +\Delta G\right)}^{2}}{4\lambda k_{B}T}\right]$$

where Δ*G* is often termed the driving force of the reaction and is the change in free energy between the final and initial states and *V* is the diabatic electron coupling matrix element between the two states. The total reorganization energy, *λ*, can be calculated from the average of the instantaneous difference in energy between the oxidized and reduced forms (Δ*E*) from both the oxidized trajectory and the reduced trajectory. Within the linear response approximation the reorganization energy is given by^56^:

(3) $$\lambda =\frac{1}{2}\left({〈\Delta E〉}_{\text{red}}-{〈\Delta E〉}_{\text{ox}}\right)$$

where ${〈\Delta E〉}_{\text{red}}$ and ${〈\Delta E〉}_{\text{ox}}$ are the average values of Δ*E* from the reduced and oxidized trajectories, respectively. The total reorganization energy has been measured to lie between 16 kcal/mol and 28 kcal/mol,^31^, ^32^, ^33^ and estimated to be about 16 kcal/mol and 23 kcal/mol from DFT and MP2 calculations.^37^ The present results for the overall average reorganization energy, from eq. (3), find *λ* =55.9 kcal/mol, which is significantly larger than the experimental value, predominantly owing to the high value for ${〈\Delta E〉}_{\text{red}}$. The accuracy of the computed value of *λ* can depend on a number of factors including applicability of the linear response approximation, the completeness of the sampling and the accuracy of the QM/MM method. The computational cost of QM/MM approaches makes it challenging to achieve a thorough sampling of configurations, and we explore the accuracy of the QM/MM model in more detail later.

**Figure 3:**
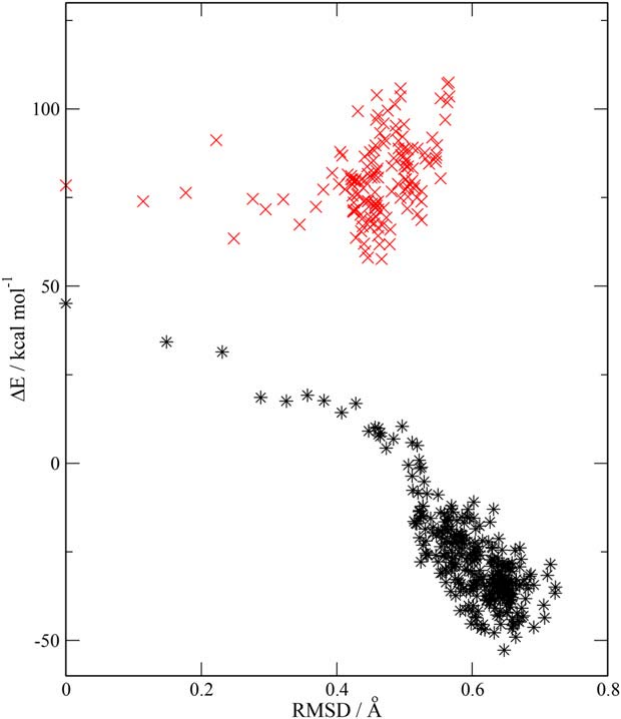
Correlation between Δ*E* and the RMSD of the protein structure relative to the structure at *t* = 0 for the QM/MM simulations. Oxidized form ∗, reduced form ×. [Color figure can be viewed at wileyonlinelibrary.com]

Figure 3 illustrates the correlation between the root mean squared deviation (RMSD) in the structure relative to the structure at *t* = 0 and the calculated Δ*E* for the oxidized and reduced trajectories. For the oxidized form, the plot suggests a significant degree of correlation between the structure and the calculated Δ*E*. There is also a large amount of change in the RMSD for the structures in the early part of the simulation (those with positive Δ*E*) confirming that the protein was still undergoing some equilibration in this period. For the reduced form there is less variation in the RMSD during the simulation, and correspondingly less variation in Δ*E*.

**Figure 4:**
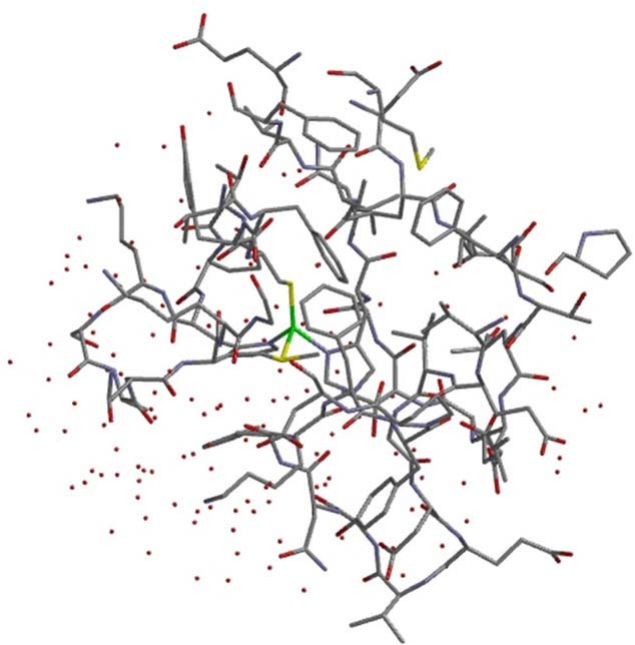
Enlarged active site region treated at the QM level, the copper atom is shown in green and the hydrogen atoms are omitted for clarity. [Color figure can be viewed at wileyonlinelibrary.com]

Assuming that the electronic coupling matrix elements do vary significantly with the fluctuations in the structure,^57^ the most efficient electron transfer will occur at the point when the reorganization energy is smallest, provided that this structure is thermally accessible. The oxidized trajectory reveals some interesting structures where the energy difference between the oxidized and reduced forms of the protein is close to zero. The value with smallest magnitude was −0.5 kcal/mol at 2.6 ps. The corresponding active site is distorted with *τ*_4_ = 0.76, and if such a structure was thermally accessible it would result in very fast rates of electron transfer. To explore the energetics of these structures further, additional calculations with a larger QM region describing the active site have been performed for the structures corresponding to the times of 2.6 ps and 24.4 ps. These structures were chosen based on their associated Δ*E*, with the structure at 2.6 ps corresponding to the minimum value and the structure at 24.4 ps close to the average value during the part of the simulation were the structure had stabilized. The larger QM region is shown in Figure 4, and represents the largest QM region that was tractable with our computational resources. This will account more accurately for structural effects further from the active site, and also has the effect of removing the point charges of the MM region from close to the copper center giving insight into the accuracy of a QM/MM partitioning in these systems. With the larger QM region the computed Δ*Ε* change from −0.5 kcal/mol to −7.0 kcal/mol at 2.6 ps and −25.0 kcal/mol to −17.7 kcal/mol at 24.4 ps. This shows that the smaller QM active site calculations correctly predict that the structure at 2.6 ps has the smaller reorganization energy. However, while the variation in the reorganization energy remains significant, it suggests that the smaller QM region tends to exaggerate these fluctuations. This is likely to be associated with having a larger number of classical point charges very close to the copper active site center.

## Conclusions

QM/MM molecular dynamics simulations have been performed for the oxidized and reduced forms of plastocyanin. The structural parameters describing the active site determined through averaging the geometries through the MD simulation are close to those of the crystal structure and show some significant differences to those of the optimized isolated gas phase active site. The strain energy for the active site is computed to be 12.8 kcal/mol for the oxidized form, in agreement with previous work.^18^ For the reduced form the strain energy is found to be 14.5 kcal/mol, which is comparable to the value for the oxidized form. This is consistent with the concept of an entatic state where the protein scaffold maintains a structure that is nonoptimal for either oxidized or reduced forms of the protein.

There is considerable variation in the computed instantaneous energy difference between the oxidized and reduced forms of the protein arising from the thermal fluctuations in the structure at room temperature. This necessitates some averaging over conformation in determining the reorganization energy, and means that the view of the protein constraining the active site in a structure that is optimal for electron transfer does not provide the complete picture of the electron transfer process. The computed value for *λ* is higher than values estimated from experiment, although the simulations do sample structures that have a much lower value for *λ*. This may indicate more extensive sampling is required. The evidence of the calculations presented here is that using a QM region that includes the typical active site treated in QM calculations reproduces the trends in the energy gap between oxidized and reduced forms, but tends to overestimate the fluctuations. This illustrates the challenge of modeling the electron transfer in these systems within a QM/MM framework. Treating a very large region of the protein surrounding the active site at the QM level is desirable for a reliable estimate of the reorganization energy. However, these calculations are very computationally demanding and are not practical for the study of many conformations or the use in molecular dynamics simulations. One solution to this problem might be to use more approximate methods, such as LFMM, provided they give a balanced treatment of both oxidized and reduced forms of the protein.
